# Fast and Accurate Classification of Meta-Genomics Long Reads With deSAMBA

**DOI:** 10.3389/fcell.2021.643645

**Published:** 2021-04-28

**Authors:** Gaoyang Li, Yongzhuang Liu, Deying Li, Bo Liu, Junyi Li, Yang Hu, Yadong Wang

**Affiliations:** ^1^Center for Bioinformatics, School of Computer Science and Technology, Harbin Institute of Technology, Harbin, China; ^2^Department of Internal Medicine, General Hospital of Heilongjiang Province Land Reclamation Bureau, Harbin, China; ^3^School of Computer Science and Technology, Harbin Institute of Technology (Shenzhen), Shenzhen, China; ^4^School of Life Science and Technology, Harbin Institute of Technology, Harbin, China

**Keywords:** long read, pseudo alignment, de Bruijn graph-based index, metagenomics 16S, read classification

## Abstract

There is still a lack of fast and accurate classification tools to identify the taxonomies of noisy long reads, which is a bottleneck to the use of the promising long-read metagenomic sequencing technologies. Herein, we propose de Bruijn graph-based Sparse Approximate Match Block Analyzer (deSAMBA), a tailored long-read classification approach that uses a novel pseudo alignment algorithm based on sparse approximate match block (SAMB). Benchmarks on real sequencing datasets demonstrate that deSAMBA enables to achieve high yields and fast speed simultaneously, which outperforms state-of-the-art tools and has many potentials to cutting-edge metagenomics studies.

## Introduction

Metagenomic sequencing is ubiquitously applied to comprehensively study environmental samples (Methé et al., 2012; Gilbert et al., 2014; Cheng et al., 2020). It enables to reveal the compositions of microbial communities in various environments and study the functions of microbial communities and their interactions to environments. Furthermore, many new species can be discovered without cultivation in laboratories. With the rapid development of high-throughput sequencing technologies, metagenomic sequencing is promising for the analysis of microbiome. Especially due to its ability of real-time and portable sequencing of the samples (Quick et al., 2016), long-read sequencing technologies have enormous potential to metagenomic studies. However, with the characteristics of long-read sequencing data, analytical challenges still remain.

In metagenomic studies, a fundamental task is to recognize the composition of the microbial community of the sequenced sample. With the ever-increasing number of sequenced genomes, it is feasible to accomplish this task by using the libraries of assembled genomes [e.g., RefSeq (Pruitt et al., 2014)] as reference to implement the taxonomy classification of sequencing reads. A common approach is to align the reads against the reference (Altschul et al., 1990; Huson et al., 2007; Cheng, 2019); however, this is not viable to handle a large amount of metagenomic reads (hundreds of gigabases) due to a low processing speed. Moreover, there are several specific technical issues in the classification of metagenomic long reads, which makes it an even more difficult computational task. Firstly, most of the long reads produced by mainstream platforms (such as ONT and PacBio platforms) are error-prone, which requires read classifiers to be noise-robust. Secondly, the reference is usually incomplete, i.e., many reads could be from unknown genomes, which requires read classifiers to handle the divergences between the sequenced genomes and their related genomes in the reference well. Thirdly, there are many common sequences among closely related genomes (e.g., various strains of bacteria species), which require read classifiers to handle the ubiquitous repeats in the reference well. Most state-of-the-art tools, such as Kraken (Wood and Salzberg, 2014; Wood et al., 2019), Centrifuge (Kim et al., 2016), Kaiju (Menzel et al., 2016), MetaOthello (Liu et al., 2018), are designed for short reads. Generally, they use pseudo alignments, i.e., the exact or approximate matches from reads to reference as signals to achieve fast speed without loss of accuracy on short reads. However, most of them rely on the assumptions on the long exact matches and/or low divergences between reads and reference, which might fail at the issues mentioned above. Long-read aligners (Li, 2018) can be used as alternatives; however, they are still time-consuming, which could not be well-suited for large-scale datasets and/or real-time tasks.

Herein, we present de Bruijn graph-based Sparse Approximate Match Block Analyzer (deSAMBA), a novel approximate match-based pseudo alignment approach for the classification of long reads. deSAMBA is motivated by the fact (Chaisson and Tesler, 2012) that sequencing errors are unevenly distributed along the reads. Many long-read aligners (Chaisson and Tesler, 2012; Li, 2013, 2018; Sedlazeck et al., 2018; Hu et al., 2019, 2020, 2021; Govindaraj et al., 2020; Hasan et al., 2020a,b) also take advantage of this model to find short exact matches (i.e., “seeds”); however, deSAMBA looks for longer approximate match blocks between reads and reference. Previous studies (Liu et al., 2017) indicate that such blocks can be specifically mapped to reference under the circumstance of sequencing noise, so it is possible for them to become noise-robust features for read classification.

## Results

### Overview of de Bruijn Graph-Based Sparse Approximate Match Block Analyzer Approach

deSAMBA is composed of some tailored designs and implementations to achieve high yields and fast speed simultaneously. Basically, it uses Unitig–Burrows–Wheeler transform (BWT) data structure (Guan et al., 2018) to index the de Bruijn graph of reference sequences and finds highly similar approximate match blocks through the index. These blocks are called sparse approximate match blocks (SAMBs), as they are usually sparsely placed along reads. Mainly, deSAMBA recognizes the taxonomy of a give read in the following four major steps.

1. deSAMBA partitions the read into a series of segments. For each of the segments, it finds the local region having highest number of consecutive k-mer matches as a seed block.
2. For each of the seeding blocks, deSAMBA retrieves a set of maximal exact matches (MEMs) to the unitigs of the reference and extends the MEMs to generate SAMBs by local alignment.
3. deSAMBA greedily merges the SAMBs and extends the merged SAMBs by sparse dynamic programming (SDP)-based pseudo alignment against local reference sequences.
4. deSAMBA scores the extended SAMBs and identifies the taxonomy of the read by the highest scored SAMB. Moreover, it also supports to output the SAMBs as pseudo alignment results.

**Figure 1:**
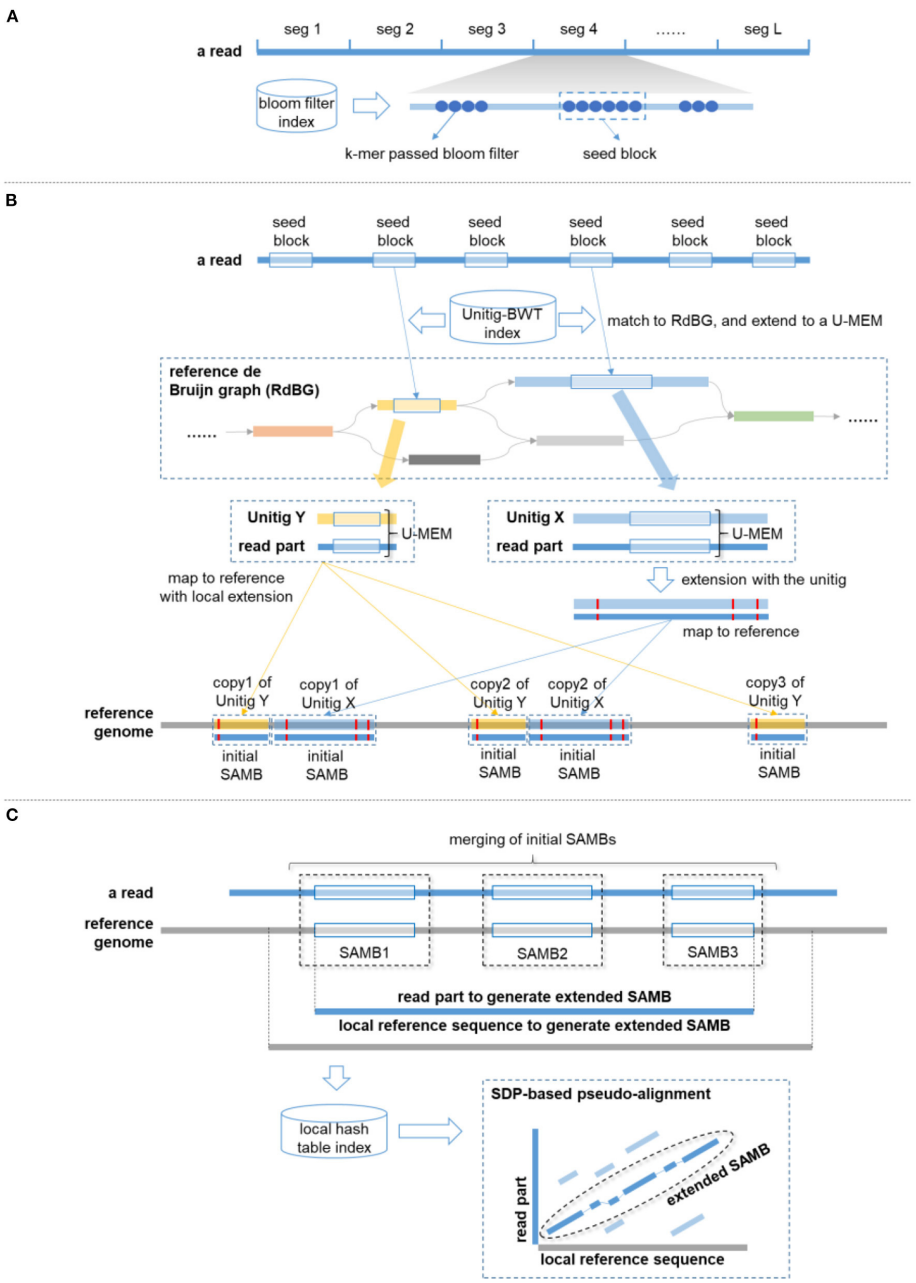
A schematics illustration of de Bruijn graph-based Sparse Approximate Match Block Analyzer (deSAMBA) approach. **(A)** The generation of seed block. A read is partitioned into fixed length segments. For a segment, all its *l*-mers (marked by blue round dots) are matched to reference through a bloom filter-based index, and the local region having the largest number of consecutive *l*-mer matches (marked by a blue dashed rectangle) is determined as a “seed block.” **(B)** The generation of initial sparse approximate match blocks (SAMBs). The seed blocks are matched to the unitigs of the RdBG through the Unitig–Burrows–Wheeler transform (BWT) index. Each of the matches is extended to a U-MEM. If a U-MEM is distanced from the two ends of the matched unitig by at least 12 bp (like the U-MEM in blue color), deSAMBA extends it to an approximate match by aligning the corresponding read part against the unitig. Further, the generated approximate match is mapped to the various copies of the unitig to be initial SAMBs (the blue unitig has 3 copies in this case). Moreover, if a U-MEM is within 12 bp of either end of the unitig (like the U-MEM in yellow color), deSAMBA maps the U-MEM to the various copies of the unitig at first (the yellow unitig has three copies in this case), and the mapped matches are as R-MEMs. For each of the R-MEMs, deSAMBA separately aligns the corresponding read part against the local reference sequence to generate a distinct initial SAMB. **(C)** The generation of extended SAMB. deSAMBA merges nearby initial SAMBs to generate a SAMB chain. In the figure, three initial SAMBs are chained, and the read part of the corresponding SAMB chain is extracted. Meanwhile, a local reference sequence is also extracted by extending the upstream and downstream boundaries of the SAMB chain on reference (1,000 bp are extended for both upstream and downstream). A hash table is then built for read to find all the short matches between the local reference and the read part. Further, a sparse dynamic programming (SDP)-based pseudo alignment is implemented between the local reference and the read part to generate an extended SAMB.

A schematics illustration is in Figure 1.

### Benchmark on Pseudo Metagenomic Datasets

We benchmarked deSAMBA with a series of pseudo metagenomic datasets and a real mock metagenome dataset. At first, we employed 145 real datasets from single genomes as pseudo metagenomic datasets (86 ONT datasets and 59 PacBio datasets; Supplementary Table 1) and implemented deSAMBA, Centrifuge, Kaiju, and Minimap2 on them. Here, 16,284 complete genomes (8,621 bacterial, 251 archaea, and 7,412 viral genomes, totaling ~35 Gbp) downloaded from NCBI RefSeq were used as reference. There are 49 ONT and 22 PacBio datasets (called “WR datasets”) whose ground truth genomes are in the reference, and for each of the remaining datasets (called “NR datasets”), there is at least one genome in the reference having common ancestry at species or genus level to its ground truth genome. The sensitivity, accuracy, F1 score, and speed of read classification were assessed. Refer to Supplementary Notes for more details about the implementation of the benchmark.

**Figure 2:**
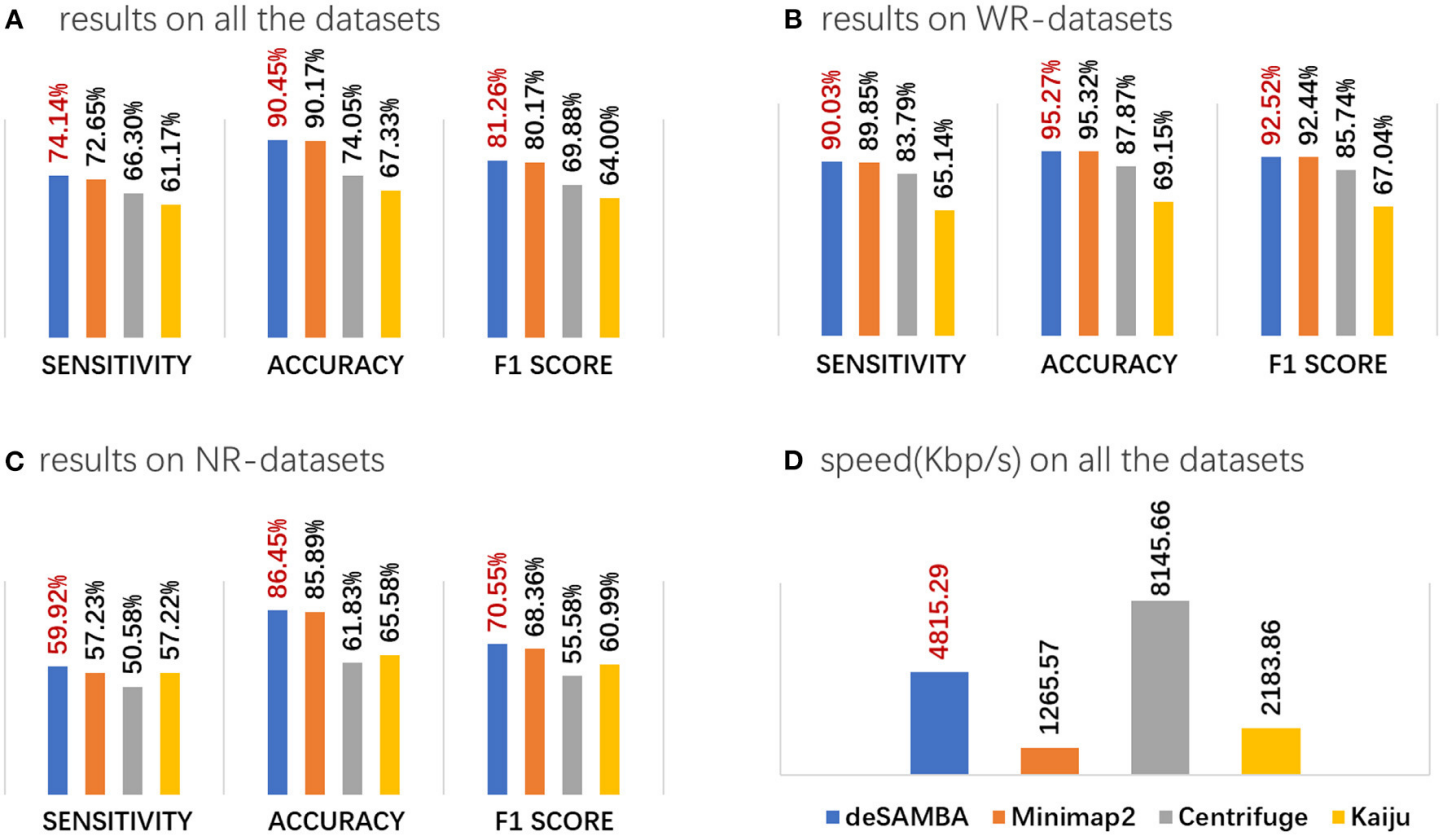
The results of various approaches on the 145 pseudo-metagenomic datasets. **(A–C)** The average sensitivity, accuracy, and F1 score on all the 145 pseudo-metagenomic datasets **(A)**, the 71 WR datasets **(B)**, and the 74 NR datasets **(C)**. The sensitivity, accuracy, and F1 score are defined as S = *N*_*TP*_/*N*_*T*_, A = *N*_*TP*_/*N*_*C*_, and F1 = 2*SA*/ (*S*+*A*), respectively, where *N*_*T*_, *N*_*C*_, and *N*_*TP*_ are, respectively, the total numbers of all the reads, the reads being classified, and the reads being correctly classified. **(D)** The speed of the approaches, which was assessed by Kbp processed per second with eight CPU threads.

Primarily, four issues are observed from the results (Figure 2, Supplementary Figure 1):

1. deSAMBA has good classification yields. deSAMBA had slightly higher F1 score than that of Minimap2 and outperformed Centrifuge and Kaiju on both of sensitivity and accuracy (Figure 2A, Supplementary Figures 1A,B). Furthermore, we assessed the classifications on the WR datasets (Figure 2B, Supplementary Figures 1C,D), and similar trends were observed. These results indicate that Centrifuge and Kaiju are easier to be affected by sequencing errors, even if the reads are from known genomes. This is mainly because such short read toward approaches rely on the assumptions of long exact matches and/or few divergences between reads and reference, which does not stand for long reads. However, as approximate matches, SAMBs are much more noise-robust, which helps find the signatures to implement a precise classification.
2. deSAMBA has good ability to classify the reads from unknown genomes. We assessed the classifications on the NR datasets (Figure 2C, Supplementary Figures 1E,F) and observed that deSAMBA also had the highest F1 score. We investigated the intermediate results and found that this is because the generated SAMBs usually had relatively large lengths and low edit distances, so that most of them were specifically mapped to the reference genomes closely related to the ground truth genomes of NR datasets. Moreover, all the approaches have reduced sensitivities and accuracies on NR datasets mainly due to the divergences between the ground truth genomes and their related genomes in the reference.
3. deSAMBA enables to identify the reads from various strains, which is an on-demand function. To assess this ability, we did an assessment with six ONT datasets having strain-level labels (lines 13, 14, 19, 24, 31, 48 of Supplementary Table 1) that a read was considered as correctly classified only if it was assigned to its ground truth strain. deSAMBA outperformed Minimaps2 slightly and Centrifuge and Kaiju significantly (Supplementary Figure 2).
4. deSAMBA has fast speed. deSAMBA is about 4 and 2 times faster than Minimap2 and Kaiju, respectively, and slower than Centrifuge (Figure 2D, Supplementary Figures 1G,H). Moreover, deSAMBA also had a nearly linear speedup with increasing number of CPU threads (Supplementary Figure 3). Besides, the memory footprint of deSAMBA is 69 GB. Comparing to that of Centrifuge (9 GB), Kaiju (31 GB), and Minimap2 (76 GB), this is acceptable, especially for modern servers.

Overall, deSAMBA achieved a better balance between yields and speed than state-of-the-art pseudo alignment-based approaches, and its fast speed more suited to large-scale datasets and real-time tasks than long-read aligners. It is also observed that deSAMBA had <80% sensitivities on seven WR datasets (Supplementary Figure 4), indicating that they were still not handled well. We further aligned those reads directly to their reference by BLASTN (Boratyn et al., 2013). The results (Supplementary Figure 5) indicated that, on average, BLASTN failed to align nearly 30% of the reads, similar to the classification results of deSAMBA. So, we realized that these datasets could have relatively poor sequencing quality, which affect the yields of deSAMBA.

### Benchmark on Real Mock Community Dataset

Further, we benchmarked deSAMBA, Minimap2, Centrifuge, Kaiju, and MetaMaps (Dilthey et al., 2019) with a real ONT dataset (SRA accession number: ERR3200811, 367173 reads and 2.36G bases in total) from a mock community [GIS20 (Bertrand et al., 2019)] that consists of 20 species with abundances range from 0.1 to 30% (Supplementary Table 2). We also composed a set of ground truth taxonomies (GTTs) for the assessment (Supplementary Table 2). That is, if a GIS20 species has its own genome in the reference library, the taxonomy ID of the genome was used as the GTT of the species; otherwise, if there are genomes in the reference library that have a common ancestry at species or genus level to the GIS20 species, the taxonomy ID of the lowest common ancestry was used as the GTT. It is also worth noting that two GIS20 species did not have GTTs, since there was no genome in the reference that has a common ancestry to them at the genus or lower level.

**Figure 3:**
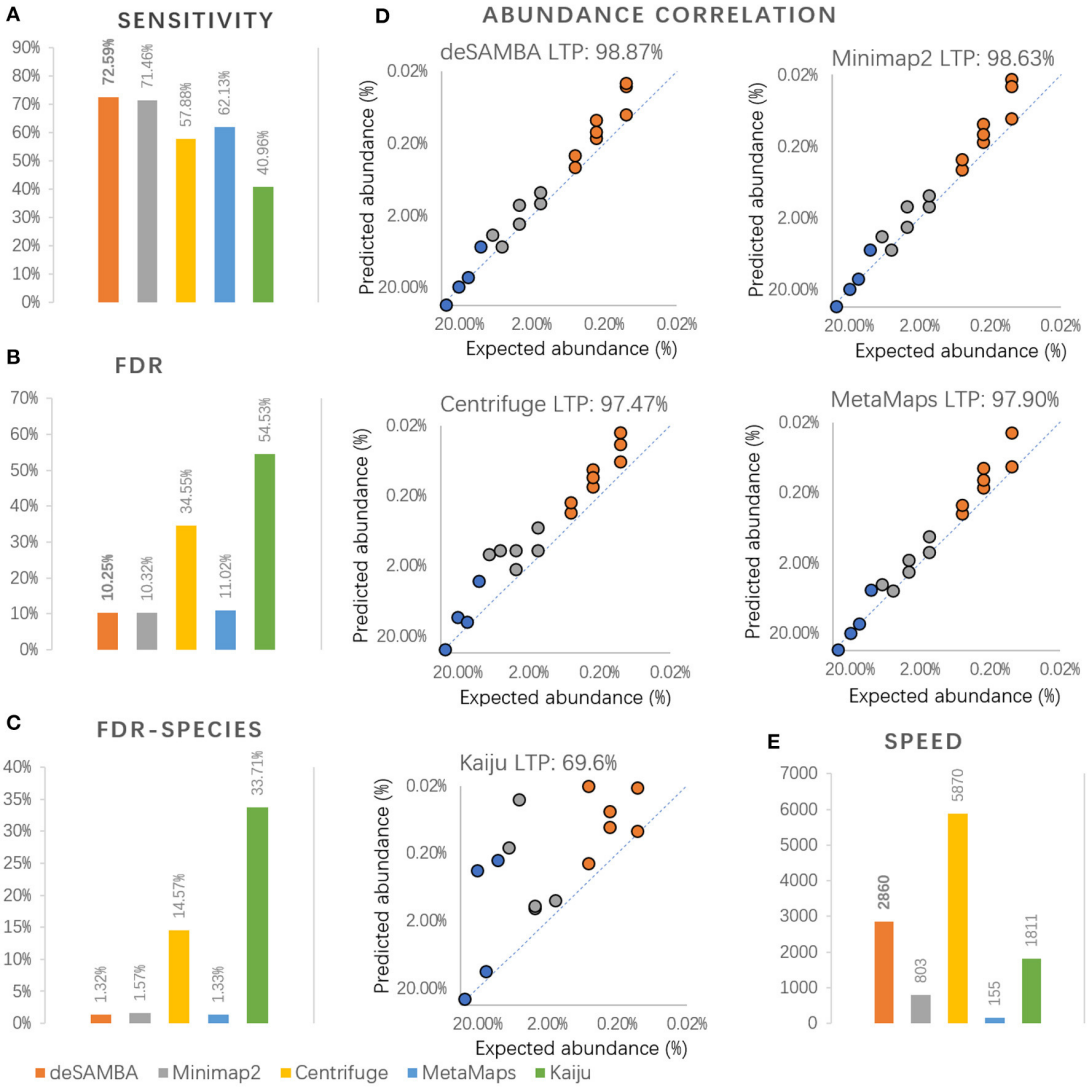
The results of various approaches on GIS20 mock metagenome dataset. **(A–C)** The sensitivity, false discovery rate (“FDR”), false discovery rate at species or higher level (“FDR-species”) of various approaches on GIS20 mock metagenome dataset. Herein, the sensitivity is defined as *N*_*G*_/*N*_*T*_, and the FDR is defined as *N*_*F*_/*N*_*C*_, where *N*_*T*_ is the total number of bases in the dataset, *N*_*G*_ is the number of bases being classified to ground truth taxonomies (GTTs), *N*_*C*_ is the number of bases being classified, and *N*_*F*_ is the number of bases being classified to non-GTT taxonomies. It is worth noting that species-level taxonomy IDs were used as GTTs instead of strain-level ones when calculating FDR-species. **(D)** Log-transformed Pearson's correlation (“LTP”) between the proportions of bases classified to GTTs and the corresponding expected abundances. It is worth noting that the proportions of 18 GTTs were used to calculate the correlations, since two of the GIS20 species did not have their GTTs in the reference. **(E)** The speed of the approaches, which was assessed by Kbp processed per second with eight CPU threads.

The sensitivities and false discovery rates (FDRs) of the approaches were assessed. The results (Figures 3A,B) indicate that deSAMBA had the highest sensitivity and lowest FDR, indicating that overall, it had the best yields on the GIS20 dataset. It is worth noting that the FDRs of all the approaches are quite high (>10%). This is mainly because 14 of the GIS20 species had strain-level GTTs, i.e., there were not only their own genomes but also other strains of the same species in the reference. Under such circumstance, the classification is considered as correct only if the bases are classified to the correct strains. However, there are ubiquitous long common sequences among the various strains of the species, so that it was extremely difficult to recognize the strain-level taxonomy entities of the reads. We further used the corresponding species-level taxonomy IDs as GTTs for the 14 GIS20 species and reevaluated the classification results and observed much lower FDRs (Figure 3C).

We further assessed the proportions of bases being classified to various GTTs. Log-transformed Pearson's correlations between the proportions of classified bases to GTTs and the corresponding expected abundances were calculated. The results (Figure 3D) suggested that deSAMBA achieved the highest correlations, indicating that its classifications mostly coincide with the ground truths of the dataset. Moreover, no large divergence was observed between the proportions of classified bases and their corresponding GTTs, which suggests that deSAMBA has the ability to handle various species well. The correlation of Kaiju was quite low mainly because it has poor ability to produce correct strain-level classifications.

The speed of the approaches was also assessed (Figure 3E), and Centrifuge was still the fastest (5,870 Kbp/s). deSAMBA (2,860 Kbp/s) was the best runner-up and the fastest long read-toward approach, i.e., about 3.5 and 18.5 times faster than that of Minimap2 (803 Kbp/s) and MetaMaps (155 Kbp/s).

## Discussion

Due to the combination of the high sequencing errors, the large sequence divergences between sequenced unknown genomes and reference genomes and the large size of reference sequences, it is still a non-trivial task to implement fast and accurate long-read classification. With the rapid growth of long-read sequencing metagenomics data, it has become a pressing need to develop more advanced computational approaches to break through this bottleneck with the use of metagenomic long reads. Herein, we present deSAMBA to show how to use SAMBs as a kind of useful signal to implement fast and accurate metagenomic read classification. Mainly, deSAMBA has three advantages to long-read classification as follows:

Firstly, as approximate matches, SAMBs enable to better handle the sequencing noise and the divergences between reference and related genomes, and this feature helps to achieve higher sensitivity than that of short read toward algorithms that usually use exact matches or only allow low divergences between reads and reference.

Secondly, as longer matches, SAMBs are feasible to handle the ubiquitous repeats in reference, and this feature helps improve the specificity of the matches and effectively narrow down the searching space during read classification, which paves the way to implement accurate classification. Moreover, the narrowed searching space also helps accelerate read classification speed.

Thirdly, deSAMBA has several tailored implementations, especially on the Unitig–BWT index and pseudo alignment method. They help to achieve high performance with moderate cost of computational resources that is affordable to modern servers and high-performance clusters (HPCs). This is well-suited to large-scale datasets and real-time tasks.

The benchmark on a series of real sequencing datasets suggests that deSAMBA improves the yields of long-read classification substantially, comparing to state-of-the-art pseudo alignment-based read classification tools. Meanwhile, deSAMBA can produce equally good classifications to state-of-the-art long aligners, while it is times faster. Considering its yields and performance, deSAMBA achieves a good balance, and it is a promising productivity tool in metagenomic data analysis. We believe that deSAMBA has enormous potential to cutting-edge metagenomic studies.

## Methods

### The Indexing of Reference Sequences

deSAMBA organizes the reference sequences in a de Bruijn graph-based approach, which is initially proposed in deBGA (Liu et al., 2016). To reduce memory use, we used Unitig–BWT data structure (Guan et al., 2018) to index the unitig of the de Bruijn graph of the given reference sequences. More precisely, deSAMBA constructs the de Bruijn graph of reference sequences at first and extracts the unitigs. The BWT is then constructed for the concatenated unitigs as the Unitig–BWT of the reference sequences. This indexing approach has good balance between retrieval speed and RAM space cost. It is also worth noting that instead of assigning a unique taxonomy ID like the previous study (Guan et al., 2018), deSAMBA maintains a position list for each of the unitigs. For a certain unitig, each item of the position list records a genomic position in reference, which represents the location of a specific copy of the unitig.

In addition to the Unitig–BWT index, deSAMBA also builds a bloom filter-based index, which is used as an auxiliary index to the generation of SAMBs. The bloom filter-based index enables to give a quick answer whether a k-mer in a given read appears in the reference, and it helps to fast find the candidate positions that are likely to be within the read blocks highly similar to local reference sequences.

With the Unitig–BWT index and the auxiliary bloom filter-based index, deSAMBA classifies a given read in the following four steps.

### The Generation of Seed Blocks

deSAMBA initially partitions a given read into 100-bp-long segments. For each of the segments, deSAMBA extracts all its l-mers and separately tests each of the l-mer with the bloom filter-based index. The l-mers that passed the bloom filter are recorded, and deSAMBA finds the local region in the segment having the most consecutive passed l-mers, i.e., the longest passed l-mer chain, as a “seed block.” It is also worth noting that the l parameter is automatically configured in advance according to the size of the reference, and it is set as 15–19 bp in most of the cases.

This idea derives from the characteristics of bloom filter. With a bloom filter-based index, there could be a proportion of false positives, i.e., a passed k-mer could be a false-positive “hit” to reference. However, the false-positive rate is relatively low, so that a seed block is more likely to be a true positive >l bp long exact match to the reference than false positives. Therefore, deSAMBA assumes that the seed blocks are long exact matches and uses them as candidates to generate SAMBs. Moreover, it is not problematic if a seed block is a false-positive match, since deSAMBA would retrieve the corresponding sequence of the seed block through the Unitig–BWT index and filter it out if no such sequence can be found.

### The Generation of Initial Sparse Approximate Match Blocks

For each of the seed blocks, deSAMBA extracts all the suffixes of the seed block and efficiently retrieves the maximal exact matches between the suffixes and the unitigs of the reference through the Unitig–BWT index. All the retrieved matches are called U-MEMs, and deSAMBA separately checks each of them. If a U-MEM is fully covered by another one, deSAMBA would filter it out. After the filtration, the longest eight remaining U-MEMs are used to generate initial SAMBs.

For each of the U-MEMs, deSAMBA checks if the match is distanced from both of the two ends of the located unitig by at least 12 bp. If so, deSAMBA uses Landau–Vishkin algorithm to compose an alignment between the read part and unitig to extend the U-MEM to a longer approximate match block. The extension is limited to the flanking 12 bp of the located unitig of the R-MEM, and it is expected that the alignment has a low edit distance, and a quality score is assigned to the generated approximate match block. The quality score is calculated based on all the matched and mismatched bases in the alignment with the following equations:

(1) $$\begin{array}{l} S=\underset{\begin{array}{c} matched\\ bases \end{array}}{\sum }S_{match}\times N_{match}+\underset{\begin{array}{c} mismatched\\ bases \end{array}}{\sum }S_{mis}\times N_{mis}\\ \;+S_{Ref\text{\_}Complex\text{\_}Penalty} \end{array}$$

(2) $$\begin{array}{l} S_{match}=-10\times \mathrm{lg}\left(\frac{0.25}{1-\text{E}}\right) \end{array}$$

(3) $$\begin{array}{l} S_{mis}=-10\times \mathrm{lg}\left(\frac{0.75}{\text{E}}\right) \end{array}$$

(4) $$\begin{array}{l} S_{Ref\text{\_}Complex\text{\_}Penalty}=-10\times \mathrm{lg}\left({\text{L}}_{reference}\right) \end{array}$$

Herein, *S*_*match*_ and *S*_*mis*_ are the scores of matched and mismatched bases, *S*_*Ref*_*Complex*_*Penalty*_ is a reference size-based penalty that is related to the total length of the reference L_*reference*_, *N*_*match*_ and *N*_*mis*_ are, respectively, the numbers of matched bases and mismatched bases in the alignment, and E is a parameter representing the expected sequencing error rate (default value: 0.15). After the extension, deSAMBA maps the alignment block to all the copies of the unitig to produce a series of “generated approximate match blocks.”

If the distance between the U-MEM is within 12 bp of either end of its located unitig, deSAMBA produces approximate match blocks in a different way. That is, deSAMBA maps the U-MEM to all the copies of its matched unitig at first, i.e., the U-MEMs are converted to one or more MEMs to local reference sequences (each of them is called an “R-MEM”). And then, deSAMBA separately extends the R-MEMs by Landau–Vishkin algorithm and scores the generated approximate match blocks in a similar approach.

After scoring, the generated approximate match blocks having >30 quality scores remained as “initial SAMBs,” and other ones are discarded. Moreover, each of the SAMBs can be written as a 4-tuple: $SAMB_{i}=\left(r_{i}^{S},r_{i}^{E},R_{i}^{S},R_{i}^{E}\right)$, where $r_{i}^{S}$ and $r_{i}^{E}$ are the start and end positions on the read, and $R_{i}^{S}$ and $R_{i}^{E}$ are the start and end positions on the reference, respectively.

### The Generation of Extended Sparse Approximate Match Blocks

deSAMBA greedily merges initial SAMBs from upstream to downstream. Two SAMBs are combined if their distance is <300 bp on the reference, and the difference between their distances on the read and on the reference is <30 bp. After this processing, the initial SAMBs are combined as a series of SAMB chains. Each SAMB chain can be written as a series of SAMBs:

(5) $$\begin{array}{l} SC_{i}=\left\{SAMB_{ij},j=1\ldots |SC_{i}|\right\} \end{array}$$

where |*SC*_*i*_| is the number of SAMBs of *SC*_*i*_. Moreover, it can be derived that the read part and the local reference sequence covered by a SAMB chain *SC*_*i*_ are $\left[r_{i1}^{S},r_{i|SC_{i}|}^{E}\right]$ and $\left[R_{i1}^{S},R_{i|SC_{i}|}^{E}\right]$, respectively.

deSAMBA further extends the SAMB chains by a SDP-based pseudo alignment approach. For a SAMB chain, *SC*_*i*_, this is done in the following four sub-steps.

1. deSAMBA extracts all the 9-mers within $\left[R_{i1}^{S}-LR,R_{i|SC_{i}|}^{E}+LR\right]$ and indexes them with a hash-table-based data structure. The parameter LR (default value: 1,000) defines an extended local region in reference.
2. deSAMBA retrieves all the 9-bp matches between $\left[r_{i1}^{S},r_{i|SC_{i}|}^{E}\right]$ and $\left[R_{i1}^{S}-LR,R_{i|SC_{i}|}^{E}+LR\right]$ through the 9-mer hash table and combines all the consecutive 9-mer matches into one or more longer exact matches.
3. The remaining matches are chained in an SDP approach with the following function:
  (6) $$f\left(Mat_{p}\right)=\max\;\left\{\underset{p>q\geq 1}{\max}\left\{f\left(Mat_{p}\right)+L\left(Mat_{p}\right)-\theta \left(p,q\right)\right\},L\left(Mat_{p}\right)\right\}$$
  (7) $$\theta \left(p,q\right)=\{\begin{array}{cc} \begin{array}{ll} 0.1\times & (\left(Mat_{p}^{R}-Mat_{q}^{R}\right)\\ \; & -\left(Mat_{p}^{r}-Mat_{q}^{r}\right)) \end{array} & if\;Mat_{p}^{R}-Mat_{q}^{R}<600\\ \infty & otherwise \end{array}$$
4. where Mat_*p*_ and Mat_*q*_ are the p-th and q-th matches (sorted by reference position) and they are not overlapped, $Mat_{p}^{R}$ and $Mat_{q}^{R}$ are their positions on the reference, $Mat_{p}^{r}$ and $Mat_{q}^{r}$ are their positions on the read, respectively; *f* (Mat_*p*_) is the scoring function for the Mat_*p*_, *L* (Mat_*p*_) is the length of Mat_*p*_, and θ (*p, q*) is a penalty for the two linked matches, Mat_*p*_ and Mat_*q*_.
5. After the SDP, the optimal chain of matches is obtained through backtracking, and it recorded as the “extended SAMB” generated based on *SC*_*i*_.

### The Classification of the Reads

deSAMBA collects all the generated extended SAMBs and sorts them by their scores calculated in the SDP process. deSAMBA then determines the primary classification of the reads by the taxonomy entity of the reference genome corresponding to the extended SAMB with highest score. The taxonomy entities of other extended SAMBs are as secondary classifications. Moreover, the SAMBs are also output as the partial pseudo alignments of the read.

### Implementation of Benchmark

All the benchmarks were carried out on a server with four Intel E7-4820 CPUs (32 cores) and 1 TB RAM running Ubuntu Linux OS. All the benchmarked classification tools were run in eight CPU threads. Some detailed information about employed reference sequences, the real sequencing datasets, and the command lines used for read classification is as follows.

We downloaded all reference sequences from NCBI RefSeq database. A genome sequence from RefSeq database was employed only if it is marked as “complete genome.” There are totally 8,621 bacterial, 251 archaea, and 7,412 viral genomes being used. The RefSeq ID and Taxonomy ID are described in “reference describe.txt.” For kaiju, the reference index was built using NCBI protein database due to its specifically designed read classification approach. We downloaded the 145 real sequencing pseudo-metagenomic datasets from NCBI Sequence Read Archive (SRA). The datasets are from various bacterial, viral, or archaeal genomes. It is also worth noting that for all the datasets, only the reads longer than 1,000 bp were used for the benchmark.

## Data Availability Statement

The original contributions presented in the study are included in the article/Supplementary Material, further inquiries can be directed to the corresponding author/s.

## Author Contributions

GL wrote the paper and did the experiments. YL and BL provided ideas of this work. YW and DL provided important suggestions of performance improvements. JL and DL revised this manuscript and guided how to do experiments. YH and YW supervised this work. All authors contributed to the article and approved the submitted version.

## Conflict of Interest

The authors declare that the research was conducted in the absence of any commercial or financial relationships that could be construed as a potential conflict of interest.
