# Supplementary material for: Fast and Accurate Classification of Meta-Genomics Long Reads With deSAMBA
Source: Front Cell Dev Biol. 2021 Apr 28;9:643645. doi: 10.3389/fcell.2021.643645 (PMC8127778; doi:10.3389/fcell.2021.643645)
Supplement: Supplementary file 1 [file Data_Sheet_1.docx]

**Fast and accurate classification of meta-genomics long reads with deSAMBA**

Gaoyang Li^1, +^, Yongzhuang Liu^1, +^, Deying Li^, 2^, Bo Liu^1^, Junyi Li^1, 3^, Yang Hu^1, *^ and Yadong Wang^1,4 *^

^1^Center for Bioinformatics, School of Computer Science and Technology, Harbin Institute of Technology, Harbin, Heilongjiang 150001, China

2 Department of Internal Medicine, General Hospital of Heilongjiang Province Land Reclamation Bureau, Harbin, China

^3^ School of Computer Science and Technology, Harbin Institute of Technology (Shenzhen), Shenzhen, Guangdong 518055, China

^4^ School of Life Science and Technology, Harbin Institute of Technology, Harbin, China

*To whom correspondence should be addressed.

^+^The authors should be regarded as Joint First Authors.

Contact: [gaoyangli@hit.edu.cn](mailto:ydliu@hit.edu.cn), [yongzhuang.liu@hit.edu.cn](mailto:yongzhuang.liu@hit.edu.cn), [bo.liu@hit.edu.cn](mailto:bo.liu@hit.edu.cn), [lijunyi@hit.edu.cn](mailto:lijunyi@hit.edu.cn), [huyang@hit.edu.cn](mailto:huyang@hit.edu.cn) and [ydwang@hit.edu.cn](mailto:ydwang@hit.edu.cn)

Table of Contents

[Supplementary Table 1. Real sequencing pseudo-metagenomics datasets 3](#_Toc66966093)

[Supplementary Table 2. The numbers and proportions of bases in GIS20 mock metagenome data being classified to ground truth by various approaches 8](#_Toc66966094)

[Supplementary Fig 1. The results of the various approaches on the pseudo metagenomics datasets 10](#_Toc66966095)

[Supplementary Fig 2. The results of the various approaches on the 6 ONT datasets with strain-level labels 11](#_Toc66966096)

[Supplementary Fig 3. The speed of deSAMBA with various numbers of CPU threads 12](#_Toc66966097)

[Supplementary Fig 4. The sensitivities and accuracies on the pseudo metagenomics datasets 13](#_Toc66966098)

[Supplementary Fig 5. The sensitivity of BLASTN on 7 WR-datasets with low sensitivity 14](#_Toc66966099)

[Supplementary Notes 15](#_Toc66966100)

[1. Implementation of benchmark 15](#_Toc66966101)

[2.1 Reference sequences used for benchmark 15](#_Toc66966102)

[2.2 Pseudo-metagenomics datasets and ground truth 15](#_Toc66966103)

[2.3 Command lines used for read classification 16](#_Toc66966104)

[2. References 17](#_Toc66966105)

1. **Real sequencing pseudo-metagenomics datasets**

| # | SRA accession^a^ | Sequencing  Platform^b^ | # of Reads^c^ | # of  Bases (Mbp)^d^ | Organism^e^ | Taxonomy  ID^f^ | Genus  ID^g^ | With  Reference^h^ |
| --- | --- | --- | --- | --- | --- | --- | --- | --- |
| 1 | DRR129651 | ONT | 116592 | 744.10 | *Vibrio litoralis DSM 17657* | 1123493 | 662 | no |
| 2 | DRR129656 | ONT | 38252 | 464.56 | *Vibrio aphrogenes* | 1891186 | 662 | no |
| 3 | DRR129658 | ONT | 125964 | 910.34 | *Vibrio algivorus* | 1667024 | 662 | no |
| 4 | DRR129662 | ONT | 132936 | 801.39 | *Vibrio rumoiensis* | 76258 | 662 | no |
| 5 | DRR131199 | ONT | 44741 | 364.29 | *Arthrobacter sp. MN05-02* | 1571833 | 1663 | no |
| 6 | DRR154113 | ONT | 270603 | 1838.80 | *Streptococcus agalactiae* | 1311 | 1301 | yes |
| 7 | DRR154115 | ONT | 46024 | 195.03 | *Streptococcus pneumoniae* | 1313 | 1301 | yes |
| 8 | DRR164908 | ONT | 33965 | 233.14 | *Leptotrichia trevisanii* | 109328 | 32067 | no |
| 9 | DRR164910 | ONT | 62626 | 273.77 | *Leptotrichia wadei* | 157687 | 32067 | no |
| 10 | DRR164912 | ONT | 145853 | 598.02 | *Leptotrichia goodfellowii* | 157692 | 32067 | no |
| 11 | DRR164915 | ONT | 26286 | 126.45 | *Leptotrichia trevisanii* | 109328 | 32067 | no |
| 12 | ERR1358778 | ONT | 33642 | 285.16 | *Escherichia coli* | 562 | 561 | yes |
| 13 | ERR1418239 | ONT | 2547 | 10.57 | *Escherichia coli K-12* | 83333 | 561 | yes |
| 14 | ERR1769944 | ONT | 10102 | 166.54 | *GlucoNObacter oxydans 621H* | 290633 | 441 | yes |
| 15 | ERR2109178 | ONT | 7908 | 114.92 | *Enterococcus faecium* | 1352 | 1350 | yes |
| 16 | ERR2195906 | ONT | 10039 | 27.90 | *Citrobacter koseri* | 545 | 544 | yes |
| 17 | ERR2259087 | ONT | 1533 | 25.10 | *Neisseria meningitidis* | 487 | 482 | yes |
| 18 | ERR2278795 | ONT | 104 | 0.68 | *Streptomyces cinnamoneus* | 53446 | 1883 | no |
| 19 | ERR2722109 | ONT | 288320 | 1919.33 | *Campylobacter jejuni subsp. Jejuni* | 32022 | 194 | yes |
| 20 | ERR2724040 | ONT | 69052 | 166.18 | *Mycobacterium tuberculosis* | 1773 | 1763 | yes |
| 21 | ERR776852 | ONT | 2312 | 18.77 | *Acinetobacter sp. ADP1* | 62977 | 469 | no |
| 22 | ERR977574 | ONT | 7202 | 48.23 | *Bacteroides fragilis* | 817 | 816 | yes |
| 23 | SRR3191595 | ONT | 29682 | 170.98 | *Agrobacterium tumefaciens* | 358 | 357 | yes |
| 24 | SRR4046732 | ONT | 4346 | 22.20 | *Xanthomonas citri pv. malvacearum* | 86040 | 338 | yes |
| 25 | SRR5117441 | ONT | 27892 | 217.97 | *Yersinia pestis* | 632 | 629 | yes |
| 26 | SRR5344353 | ONT | 2680 | 14.84 | *Akkermansia muciniphila* | 239935 | 239934 | yes |
| 27 | SRR5344354 | ONT | 21368 | 50.89 | *LachNOclostridium sp. An76* | 1965654 | 1506553 | no |
| 28 | SRR5344355 | ONT | 17954 | 44.43 | *Anaerotignum lactatifermentans* | 160404 | 2039240 | no |
| 29 | SRR5344357 | ONT | 5455 | 34.66 | *Bacteroides sp. An51A* | 1965640 | 816 | no |
| 30 | SRR5344358 | ONT | 15973 | 56.64 | *Flavonifractor sp. An10* | 1965537 | 946234 | no |
| 31 | SRR5429682 | ONT | 9174 | 68.80 | *Klebsiella pneumoniae subsp. Pneumoniae* | 72407 | 570 | yes |
| 32 | SRR5457530 | ONT | 6139 | 36.21 | *Clostridioides difficile* | 1496 | 1870884 | yes |
| 33 | SRR5514549 | ONT | 12219 | 72.91 | *Achromobacter denitrificans* | 32002 | 222 | yes |
| 34 | SRR5629775 | ONT | 68851 | 808.43 | *Klebsiella oxytoca* | 571 | 570 | yes |
| 35 | SRR5629779 | ONT | 6948 | 89.05 | *Serratia marcescens* | 615 | 613 | yes |
| 36 | SRR5665595 | ONT | 61681 | 836.52 | *Klebsiella pneumoniae* | 573 | 570 | yes |
| 37 | SRR5891470 | ONT | 19378 | 237.24 | *Acinetobacter baumannii* | 470 | 469 | yes |
| 38 | SRR5997379 | ONT | 9345 | 33.01 | *Mesoplasma chauliocola* | 216427 | 46239 | yes |
| 39 | SRR6082028 | ONT | 23223 | 228.70 | *Mesoplasma lactucae ATCC 49193* | 81460 | 46239 | yes |
| 40 | SRR6129218 | ONT | 93588 | 783.29 | *Mesoplasma florum* | 2151 | 46239 | yes |
| 41 | SRR6224382 | ONT | 21302 | 177.54 | *Mesoplasma entomophilum* | 2149 | 46239 | yes |
| 42 | SRR6312194 | ONT | 23297 | 49.53 | *Paenibacillus pasadenensis* | 217090 | 44249 | no |
| 43 | SRR6327830 | ONT | 25654 | 155.58 | *Legionella sainthelensi* | 28087 | 445 | yes |
| 44 | SRR6364637 | ONT | 133722 | 363.43 | *Mesoplasma syrphidae* | 225999 | 46239 | yes |
| 45 | SRR6471048 | ONT | 20039 | 383.52 | *Mycoplasma hominis* | 2098 | 2093 | yes |
| 46 | SRR6475285 | ONT | 45031 | 644.93 | *Streptomyces lunaelactis* | 1535768 | 1883 | no |
| 47 | SRR6780924 | ONT | 33201 | 165.87 | *Fusobacterium periodonticum* | 860 | 848 | yes |
| 48 | SRR6830111 | ONT | 17411 | 105.82 | *Fusobacterium nucleatum subsp. nucleatum* | 76856 | 848 | yes |
| 49 | SRR6917534 | ONT | 18610 | 121.69 | *Streptococcus pyogenes* | 1314 | 1301 | yes |
| 50 | SRR7119552 | ONT | 55045 | 332.69 | *Acinetobacter NOsocomialis* | 106654 | 469 | yes |
| 51 | SRR7119560 | ONT | 42530 | 308.59 | *Acinetobacter pittii* | 48296 | 469 | yes |
| 52 | SRR7467298 | ONT | 265157 | 2370.25 | *AmiNObacter sp. MSH1* | 374606 | 31988 | no |
| 53 | SRR7467447 | ONT | 445670 | 2554.30 | *Streptomyces sp.* | 1931 | 1883 | no |
| 54 | SRR7532470 | ONT | 58457 | 664.97 | *Clostridium botulinum* | 1491 | 1485 | yes |
| 55 | SRR7739756 | ONT | 70760 | 685.10 | *Staphylococcus aureus* | 1280 | 1279 | yes |
| 56 | SRR7908033 | ONT | 46226 | 1292.88 | *Bordetella pertussis* | 520 | 517 | yes |
| 57 | SRR7957428 | ONT | 364969 | 968.02 | *CapNOcytophaga canimorsus* | 28188 | 1016 | yes |
| 58 | SRR7989235 | ONT | 108976 | 839.33 | *Borrelia miyamotoi* | 47466 | 138 | yes |
| 59 | SRR8030961 | ONT | 25668 | 171.13 | *Microbacterium foliorum* | 104336 | 33882 | no |
| 60 | SRR8069226 | ONT | 275794 | 2176.62 | *Thalassotalea euphylliae* | 1655234 | 1518149 | no |
| 61 | SRR8081954 | ONT | 9451 | 76.11 | *SiNOrhizobium meliloti* | 382 | 28105 | yes |
| 62 | SRR8112132 | ONT | 201287 | 2248.28 | *Achromobacter sp. B7* | 2282475 | 222 | no |
| 63 | SRR8113455 | ONT | 2,174,450 | 11400.00 | *Thermoanaerobacter ethaNOlicus JW 200* | 509192 | 1754 | no |
| 64 | SRR8115246 | ONT | 2,404,454 | 6100.00 | *Pantoea agglomerans* | 549 | 53335 | yes |
| 65 | SRR8185373 | ONT | 39767 | 545.95 | *Rhodococcus sp. P1Y* | 1302308 | 1827 | no |
| 66 | SRR8278838 | ONT | 110157 | 268.48 | *Helicobacter pylori* | 210 | 209 | yes |
| 67 | SRR8306005 | ONT | 75727 | 857.01 | *Wolbachia endosymbiont of Drosophila ananassae* | 307502 | 953 | no |
| 68 | SRR8335319 | ONT | 84212 | 1092.09 | *Vibrio campbellii DS40M4* | 1088888 | 662 | yes |
| 69 | SRR8362629 | ONT | 168429 | 767.51 | *Mannheimia varigena* | 85404 | 75984 | no |
| 70 | SRR8428672 | ONT | 159650 | 1122.23 | *Ochrobactrum intermedium* | 94625 | 528 | no |
| 71 | SRR8457080 | ONT | 8417 | 43.56 | *Neisseria goNOrrhoeae* | 485 | 482 | yes |
| 72 | SRR8467877 | ONT | 525599 | 2633.69 | *Caldicellulosiruptor changbaiensis* | 1222016 | 44000 | no |
| 73 | SRR8480439 | ONT | 69399 | 918.04 | *Brevundimonas diminuta* | 293 | 41275 | no |
| 74 | SRR8480530 | ONT | 234432 | 2365.95 | *Bacillus subtilis subsp. spizizenii ATCC 6633* | 96241 | 1386 | yes |
| 75 | SRR8494918 | ONT | 100105 | 1099.82 | *Citrobacter gillenii* | 67828 | 544 | no |
| 76 | SRR8536147 | ONT | 1,000,846 | 8300.00 | *Halomonas olivaria* | 390919 | 2745 | no |
| 77 | SRR8538950 | ONT | 64431 | 173.74 | *Staphylococcus pseudintermedius* | 283734 | 1279 | yes |
| 78 | SRR8549412 | ONT | 74931 | 429.56 | *Halomonas sulfidaeris* | 115553 | 2745 | no |
| 79 | SRR8550983 | ONT | 81829 | 749.91 | *Psychrobacter sp. KH172YL61* | 2517899 | 497 | no |
| 80 | SRR8554089 | ONT | 76024 | 510.07 | *Halomonas axialensis* | 115555 | 2745 | no |
| 81 | SRR8560598 | ONT | 11084 | 165.31 | *Sphingomonas paucimobilis* | 13689 | 13687 | no |
| 82 | SRR8664470 | ONT | 543832 | 4410.84 | *Klebsiella sp. PO552* | 1972757 | 570 | no |
| 83 | SRR8697008 | ONT | 19085 | 303.34 | *Brevundimonas naejangsanensis* | 588932 | 41275 | yes |
| 84 | SRR8749593 | ONT | 13605 | 25.91 | *Burkholderia pseudomallei* | 28450 | 32008 | yes |
| 85 | SRR8767485 | ONT | 2029 | 28.51 | *Enterobacter hormaechei* | 158836 | 547 | no |
| 86 | SRR8767486 | ONT | 6602 | 100.54 | *Citrobacter freundii* | 546 | 544 | yes |
| 87 | ERR1140956 | PacBio | 64922 | 823.64 | *Staphylococcus aureus* | 1280 | 1279 | yes |
| 88 | ERR1354168 | PacBio | 100569 | 1257.47 | *Streptococcus sobrinus* | 1310 | 1301 | no |
| 89 | ERR1543212 | PacBio | 83955 | 1111.02 | *Acinetobacter junii* | 40215 | 469 | yes |
| 90 | ERR1599942 | PacBio | 11808 | 87.44 | *Campylobacter upsaliensis* | 28080 | 194 | no |
| 91 | ERR1802424 | PacBio | 88061 | 914.95 | *Actinomyces slackii* | 52774 | 1654 | no |
| 92 | ERR1805700 | PacBio | 65521 | 747.50 | *Mycoplasma hyorhinis* | 2100 | 2093 | yes |
| 93 | ERR1857497 | PacBio | 35380 | 396.41 | *Bartonella grahamii* | 33045 | 773 | no |
| 94 | ERR1883080 | PacBio | 115549 | 1013.75 | *Helicobacter mustelae* | 217 | 209 | no |
| 95 | ERR1940980 | PacBio | 82137 | 950.67 | *Helicobacter pullorum* | 35818 | 209 | no |
| 96 | ERR1940990 | PacBio | 95528 | 912.78 | *Clostridium paraputrificum* | 29363 | 1485 | no |
| 97 | ERR2125654 | PacBio | 84110 | 859.20 | *Kocuria rosea* | 1275 | 57493 | no |
| 98 | ERR2125846 | PacBio | 86844 | 1282.82 | *Achromobacter xylosoxidans* | 85698 | 222 | yes |
| 99 | ERR2146854 | PacBio | 110931 | 1257.07 | *Citrobacter youngae* | 133448 | 544 | no |
| 100 | ERR2146855 | PacBio | 89496 | 827.10 | *Actinobacillus equuli* | 718 | 713 | no |
| 101 | ERR2225503 | PacBio | 46696 | 551.10 | *Helicobacter fennelliae* | 215 | 209 | no |
| 102 | ERR2237784 | PacBio | 16373 | 123.42 | *Campylobacter ureolyticus* | 827 | 194 | no |
| 103 | ERR2237840 | PacBio | 115641 | 1343.08 | *Helicobacter cinaedi* | 213 | 209 | no |
| 104 | ERR2246641 | PacBio | 82270 | 782.65 | *Actinomyces howellii* | 52771 | 1654 | no |
| 105 | ERR2246671 | PacBio | 119321 | 1355.07 | *Shigella boydii* | 621 | 620 | no |
| 106 | ERR2432878 | PacBio | 56894 | 561.80 | *Acinetobacter calcoaceticus* | 471 | 469 | yes |
| 107 | ERR2532027 | PacBio | 79831 | 870.43 | *Neisseria gonorrhoeae* | 485 | 482 | yes |
| 108 | ERR2532043 | PacBio | 71229 | 518.42 | *Listeria innocua* | 1642 | 1637 | no |
| 109 | ERR2532096 | PacBio | 33466 | 299.86 | *Clostridium tertium* | 1559 | 1485 | no |
| 110 | ERR2532135 | PacBio | 121764 | 1175.98 | *Vibrio furnissii* | 29494 | 662 | no |
| 111 | ERR2532412 | PacBio | 84519 | 862.70 | *Actinobacillus pleuropneumoniae* | 715 | 713 | no |
| 112 | ERR2543055 | PacBio | 126847 | 1260.59 | *Shewanella putrefaciens* | 24 | 22 | no |
| 113 | ERR2749075 | PacBio | 102245 | 980.09 | *Mycobacteroides abscessus* | 36809 | 1763 | yes |
| 114 | ERR772459 | PacBio | 81190 | 894.16 | *Citrobacter koseri* | 545 | 544 | yes |
| 115 | ERR772462 | PacBio | 139046 | 922.92 | *Actinobacillus ureae* | 723 | 713 | no |
| 116 | ERR841690 | PacBio | 81618 | 1040.88 | *Neisseria lactamica* | 486 | 482 | yes |
| 117 | ERR910531 | PacBio | 152267 | 1110.66 | *Streptococcus sanguinis* | 1305 | 1301 | no |
| 118 | SRR2163073 | PacBio | 126276 | 1203.68 | *Bacillus licheniformis* | 1402 | 1386 | yes |
| 119 | SRR2560502 | PacBio | 57657 | 140.32 | *Bacteroides vulgatus* | 821 | 816 | no |
| 120 | SRR2925022 | PacBio | 117636 | 822.15 | *Clostridium botulinum* | 1491 | 1485 | yes |
| 121 | SRR4176232 | PacBio | 177858 | 704.60 | *Carnobacterium iners* | 1073423 | 2747 | no |
| 122 | SRR4180946 | PacBio | 136430 | 608.67 | *Pseudarthrobacter chlorophenolicus* | 85085 | 1742993 | no |
| 123 | SRR4181438 | PacBio | 29049 | 131.32 | *Rhodococcus percolatus* | 45461 | 1827 | no |
| 124 | SRR4181588 | PacBio | 236611 | 802.53 | *Amycolatopsis niigatensis* | 369932 | 1813 | no |
| 125 | SRR4236930 | PacBio | 111645 | 474.70 | *Achromobacter sp. MFA1 R4* | 1881016 | 222 | no |
| 126 | SRR4237132 | PacBio | 231416 | 869.52 | *Terriglobus roseus* | 392734 | 392733 | no |
| 127 | SRR4244870 | PacBio | 118334 | 493.82 | *Bradyrhizobium erythrophlei* | 1437360 | 374 | no |
| 128 | SRR5165186 | PacBio | 43375 | 88.08 | *Mesorhizobium loti* | 381 | 68287 | no |
| 129 | SRR5165459 | PacBio | 167285 | 630.72 | *Mesorhizobium australicum* | 536018 | 68287 | no |
| 130 | SRR5320243 | PacBio | 44474 | 146.97 | *Mycoplasma hyopneumoniae* | 2099 | 2093 | yes |
| 131 | SRR5829900 | PacBio | 145413 | 619.16 | *Clostridium beijerinckii* | 1520 | 1485 | yes |
| 132 | SRR6256365 | PacBio | 104731 | 462.71 | *Caldicellulosiruptor bescii* | 31899 | 44000 | no |
| 133 | SRR7072821 | PacBio | 160498 | 640.46 | *Halanaerobium congolense* | 54121 | 2330 | no |
| 134 | SRR7135411 | PacBio | 69488 | 378.16 | *Acidovorax citrulli* | 80869 | 12916 | yes |
| 135 | SRR7637788 | PacBio | 105804 | 401.74 | *Helicobacter saguini* | 1548018 | 209 | no |
| 136 | SRR7967879 | PacBio | 92278 | 1108.33 | *Borreliella burgdorferi* | 139 | 64895 | yes |
| 137 | SRR8175749 | PacBio | 65808 | 1006.98 | *Acinetobacter haemolyticus* | 29430 | 469 | yes |
| 138 | SRR8270580 | PacBio | 254965 | 597.83 | *Xanthomonas vasicola* | 56459 | 338 | no |
| 139 | SRR8281303 | PacBio | 119161 | 844.92 | *Deinococcus wulumuqiensis* | 980427 | 1298 | no |
| 140 | SRR8292561 | PacBio | 93819 | 1382.05 | *Salmonella enterica* | 28901 | 590 | yes |
| 141 | SRR8293937 | PacBio | 215678 | 1066.08 | *Streptococcus agalactiae* | 1311 | 1301 | yes |
| 142 | SRR8436601 | PacBio | 31434 | 66.13 | *Escherichia coli* | 562 | 561 | yes |
| 143 | SRR8476224 | PacBio | 65455 | 416.61 | *Bacillus coagulans* | 1398 | 1386 | yes |
| 144 | SRR8494910 | PacBio | 145862 | 1239.14 | *Citrobacter braakii* | 57706 | 544 | yes |
| 145 | SRR8691672 | PacBio | 48351 | 441.98 | *Acinetobacter baumannii* | 470 | 469 | yes |

a) The SRA accession number of the dataset;

b) The type of sequencing platform produces the dataset (ONT or PacBio);

c) The number of reads in the dataset;

d) The total number of bases in the dataset, counted by million base-pairs (Mbp);

e) The organism of the sample of the dataset, i.e., the ground truth genome the dataset being from;

f) The taxonomy ID of the organism of the dataset;

g) The genus ID of the organism of the dataset;

h) Whether the ground truth genome sequence is in the reference.

1. **The numbers and proportions of bases in GIS20 mock metagenome data being classified to ground truth by various approaches**

| **#** | **Species** | | **Expected**  **Number** | **deSAMBA**  **(s = 64)** | **deSAMBA**  **(s = 100)** | **Minimap2** | **Centrifuge** | **MetaMaps** | **Kaiju** |
| --- | --- | --- | --- | --- | --- | --- | --- | --- | --- |
| 1 | *Pseudomonas putida* | #Base | 707875994 * | 649935867 | 632206298 | 636651282 | 637265139 | 554082522 | 616737687 |
|  |  | #Proportion | 30.00% | 33.88% | 34.10% | 33.86% | 30.54% | 33.63% | 29.01% |
| 2 | *Klebsiella pneumoniae* | #Base | 471917329 | 414631856 | 405186764 | 407790936 | 392899870 | 360699720 | 307235418 |
|  |  | #Proportion | 20.00% | 21.61% | 21.86% | 21.69% | 18.83% | 21.89% | 14.45% |
| 3 | *Acinetobacter baumannii* | #Base | 353937997 | 275003070 | 268734026 | 269816013 | 266099707 | 237087355 | 250599732 |
|  |  | #Proportion | 15.00% | 14.33% | 14.50% | 14.35% | 12.75% | 14.39% | 11.79% |
| 4 | *Staphylococcus epidermidis* | #Base | 235958665 | 134713818 | 130686868 | 131034672 | 128889716 | 109135345 | 116607313 |
|  |  | #Proportion | 10.00% | 7.02% | 7.05% | 6.97% | 6.18% | 6.62% | 5.49% |
| 5 | *Enterococcus faecium* | #Base | 165171065 | 149642289 | 146482755 | 146791137 | 142619804 | 128217266 | 131869161 |
|  |  | #Proportion | 7.00% | 7.80% | 7.90% | 7.81% | 6.83% | 7.78% | 6.20% |
| 6 | *Salmonella enterica* | #Base | 117979332 | 118809737 | 116244384 | 116892301 | 110718105 | 104690255 | 84166214 |
|  |  | #Proportion | 5.00% | 6.19% | 6.27% | 6.22% | 5.31% | 6.35% | 3.96% |
| 7 | *Neisseria subflava* | #Base | 70787599 | 27560799 | 22433127 | 25419687 | 25043719 | 30041423 | 28685625 |
|  |  | #Proportion | 3.00% | 1.44% | 1.21% | 1.35% | 1.20% | 1.82% | 1.35% |
| 8 | *Capnocytophaga ochracea* | #Base | 70787599 | 51655150 | 49977669 | 50210350 | 47582411 | 44378401 | 42395142 |
|  |  | #Proportion | 3.00% | 2.69% | 2.70% | 2.67% | 2.28% | 2.69% | 1.99% |
| 9 | *Lactobacillus reuteri* | #Base | 35393800 | 26769371 | 25436570 | 25880118 | 26540805 | 22839983 | 24726039 |
|  |  | #Proportion | 1.50% | 1.40% | 1.37% | 1.38% | 1.27% | 1.39% | 1.16% |
| 10 | *Bifidobacterium longum *** | #Base | 35393800 | 21216422 | 20542070 | 20659268 | 19813448 | 17673266 | 17966641 |
|  |  | #Proportion | 1.50% | 1.11% | 1.11% | 1.10% | 0.95% | 1.07% | 0.85% |
| 11 | *Finegoldia magna* | #Base | 11797933 | 8346920 | 8033284 | 8098295 | 7314086 | 6542420 | 6094047 |
|  |  | #Proportion | 0.50% | 0.44% | 0.43% | 0.43% | 0.35% | 0.40% | 0.29% |
| 12 | *Enterobacter cloacae* | #Base | 11797933 | 7903827 | 7749626 | 6337493 | 8178528 | 5308735 | 27375818 |
|  |  | #Proportion | 0.50% | 0.41% | 0.42% | 0.34% | 0.39% | 0.32% | 1.29% |
| 13 | *Bifidobacterium adolescentis *** | #Base | 5898967 | 1903008 | 1815155 | 1827416 | 1837073 | 1491033 | 1777403 |
|  |  | #Proportion | 0.25% | 0.10% | 0.10% | 0.10% | 0.09% | 0.09% | 0.08% |
| 14 | *Streptococcus parasanguinis* | #Base | 5898967 | 3517483 | 3383509 | 3440288 | 4888071 | 3189656 | 6422237 |
|  |  | #Proportion | 0.25% | 0.18% | 0.18% | 0.18% | 0.23% | 0.19% | 0.30% |
| 15 | *Fusobacterium nucleatum* | #Base | 5898967 | 2717536 | 2532533 | 2551595 | 3149608 | 2196184 | 3027116 |
|  |  | #Proportion | 0.25% | 0.14% | 0.14% | 0.14% | 0.15% | 0.13% | 0.14% |
| 16 | *Prevotella oris* | #Base | 2359587 | 624475 | 238978 | 434560 | 773701 | 1392180 | 2027005 |
|  |  | #Proportion | 0.10% | 0.03% | 0.01% | 0.02% | 0.04% | 0.08% | 0.10% |
| 17 | *Helicobacter cinaedi* | #Base | 2359587 | 1659273 | 1627792 | 2383058 | 2501730 | 1500918 | 2308706 |
|  |  | #Proportion | 0.10% | 0.09% | 0.09% | 0.13% | 0.12% | 0.09% | 0.11% |
| 18 | *Parascardovia denticolens* | #Base | 2359587 | 571254 | 524676 | 537499 | 513790 | 456371 | 464343 |
|  |  | #Proportion | 0.10% | 0.03% | 0.03% | 0.03% | 0.02% | 0.03% | 0.02% |

Description：

* ：All results are based on GENUS level taxonomy.

** ：*Bifidobacterium longum* and *Bifidobacterium adolescentis* and are from same GENUS, so their results are based on species level taxonomy.


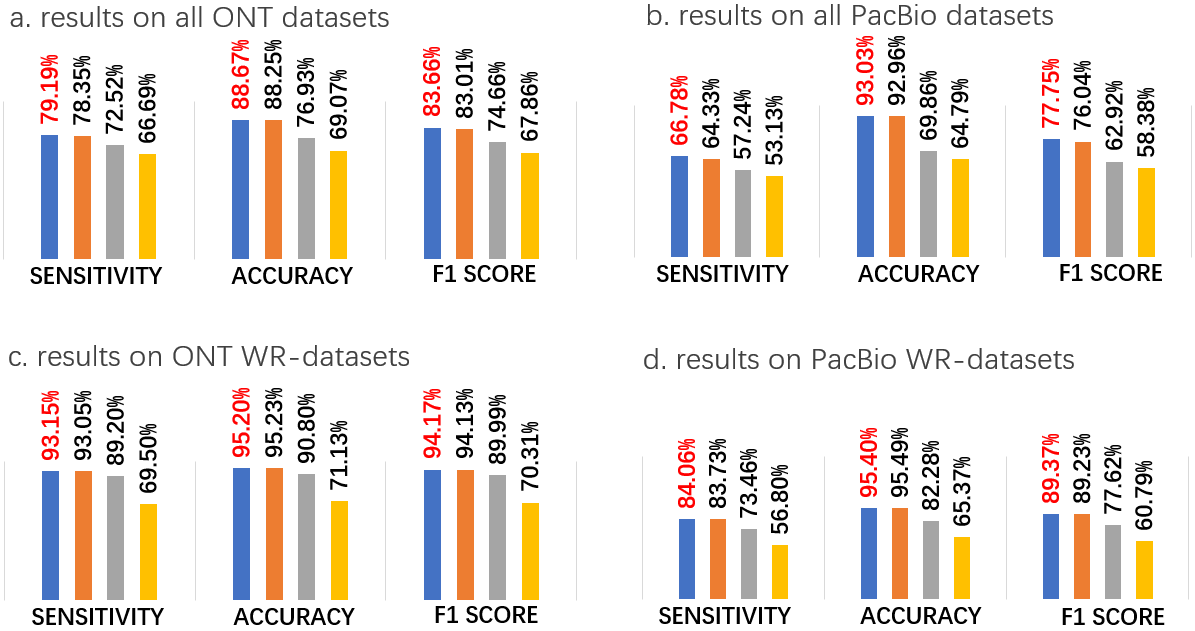


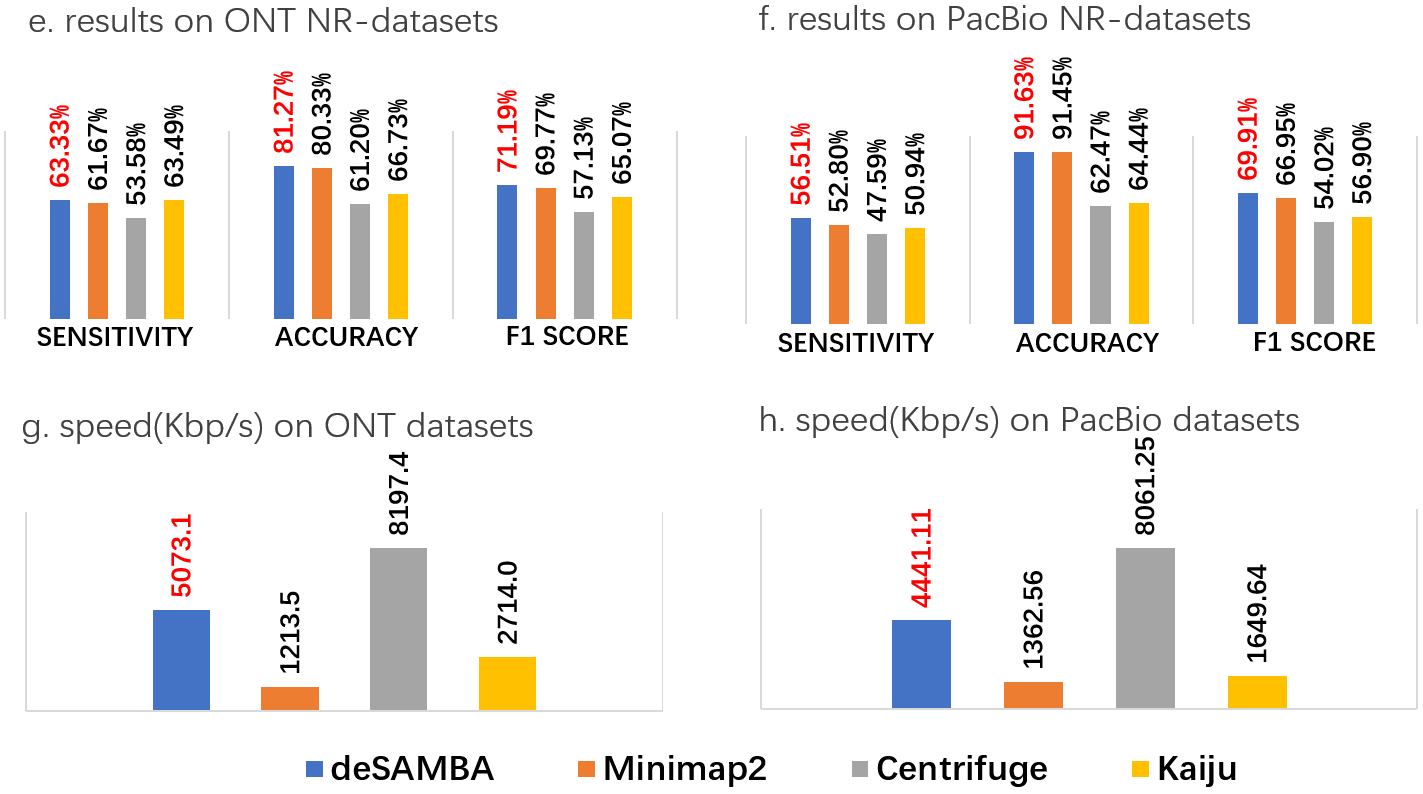


1. **The results of the various approaches on the pseudo metagenomics datasets**

The subfigures a-f respectively indicate the average sensitivity, accuracy, F1-score on all the 86 ONT (a) and 59 PacBio (b) datasets, the WR-datasets produced by ONT (c) and PacBio (d) platforms, the NR-datasets produced by ONT (e) and PacBio (f) platforms. The speed of the various approaches on all the 86 ONT and 59 PacBio datasets are respectively shown in subfigures g and h (assessed with 8 CPU threads).


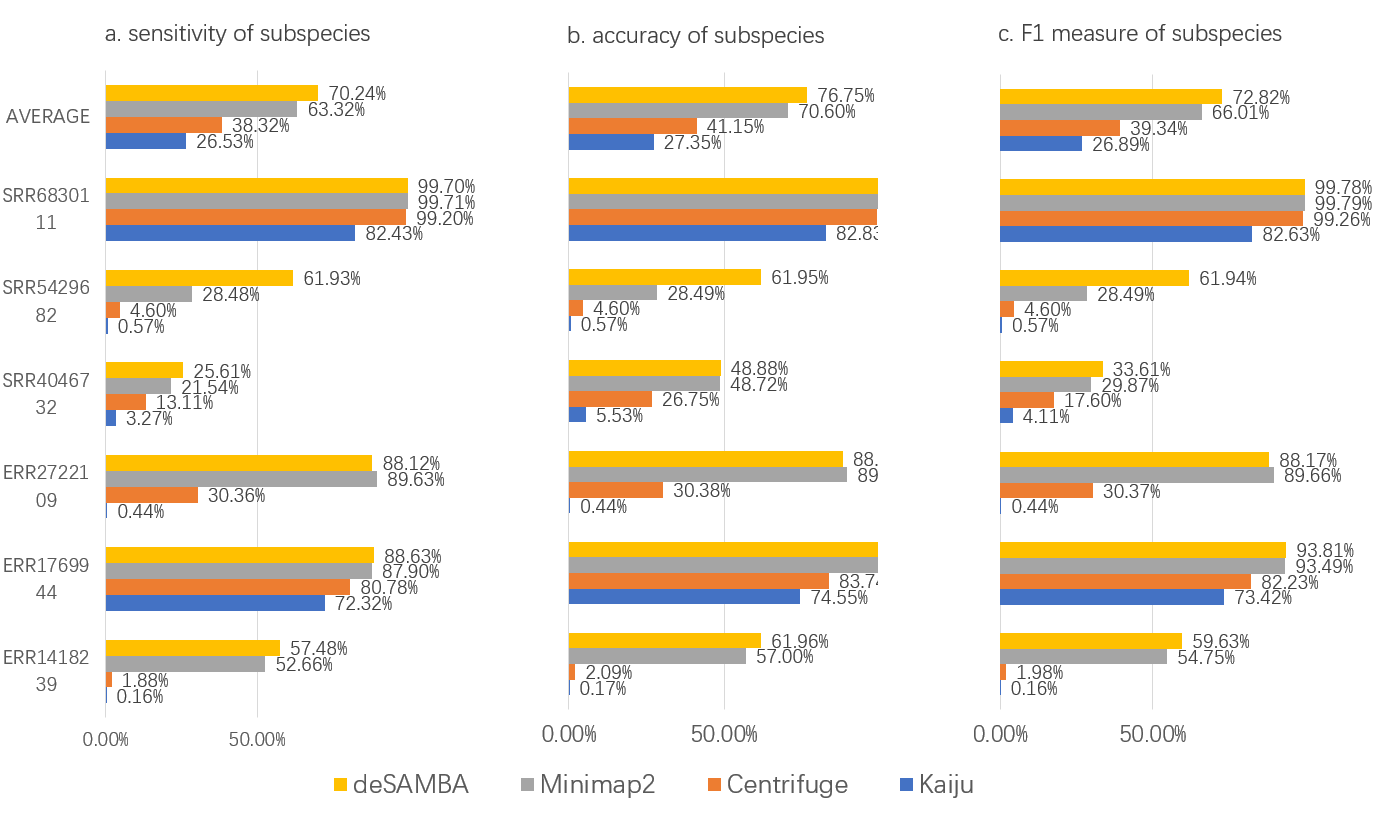


1. **The results of the various approaches on the 6 ONT datasets with strain-level labels**

The sensitivity (a), accuracy (b) and F1-score (c) of the four approaches on the 6 ONT datasets with strain-level labels. The averaged statistics (marked as “AVERAGE”) and the statistics on the 6 datasets (SRA Accessions: ERR1418239, ERR1769944, ERR2722109, SRR4046732, SRR5429682 and SRR6830111, corresponding to lines 13, 14, 19, 24, 31, 48 of Supplementary Table 1) are respectively shown in the bar plots.


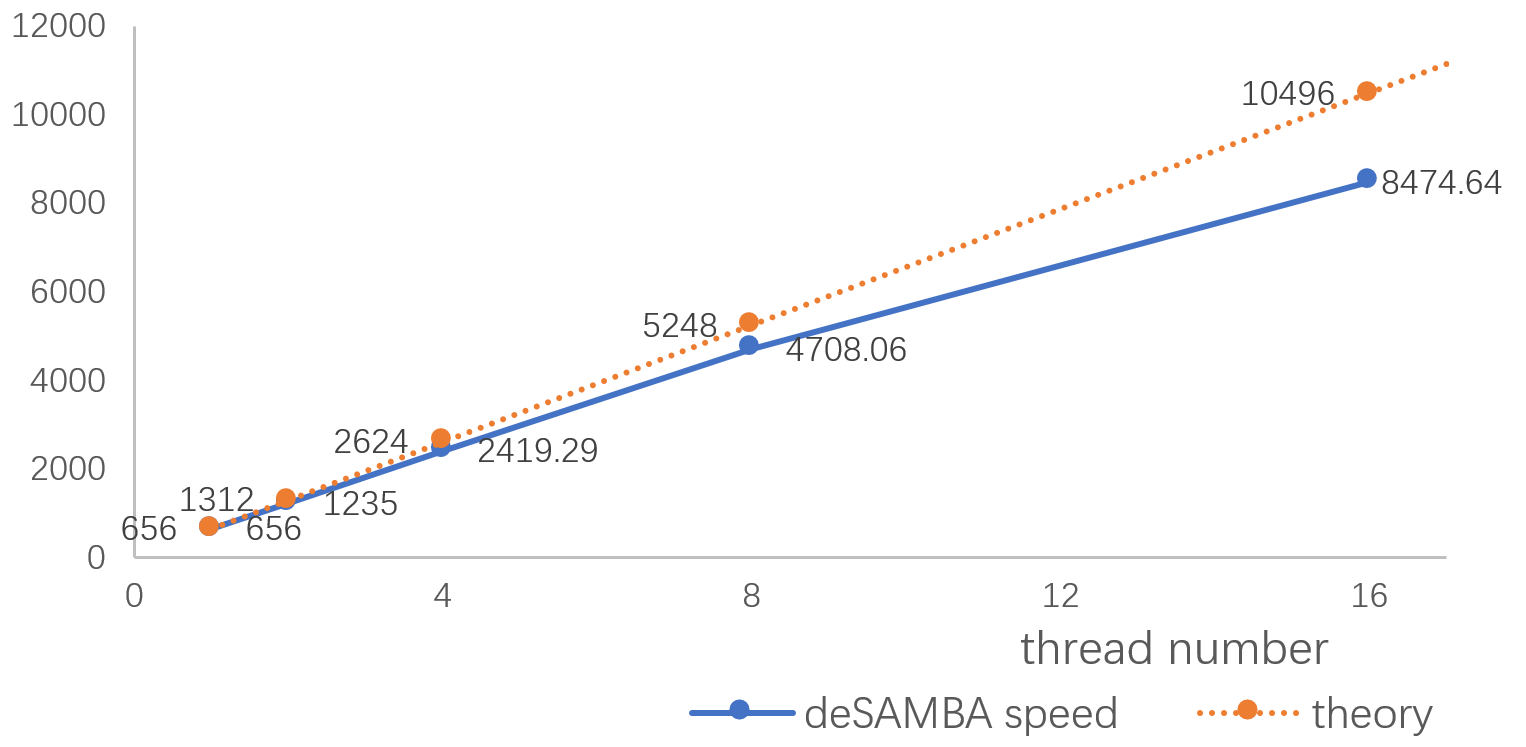


1. **The speed of deSAMBA with various numbers of CPU threads**

The figure shows the speed of deSAMBA with 1, 2, 4, 8 and 16 CPU threads (blue line and dots). The orange dashed line and dots indicate the speedup in theory with the same numbers of threads.


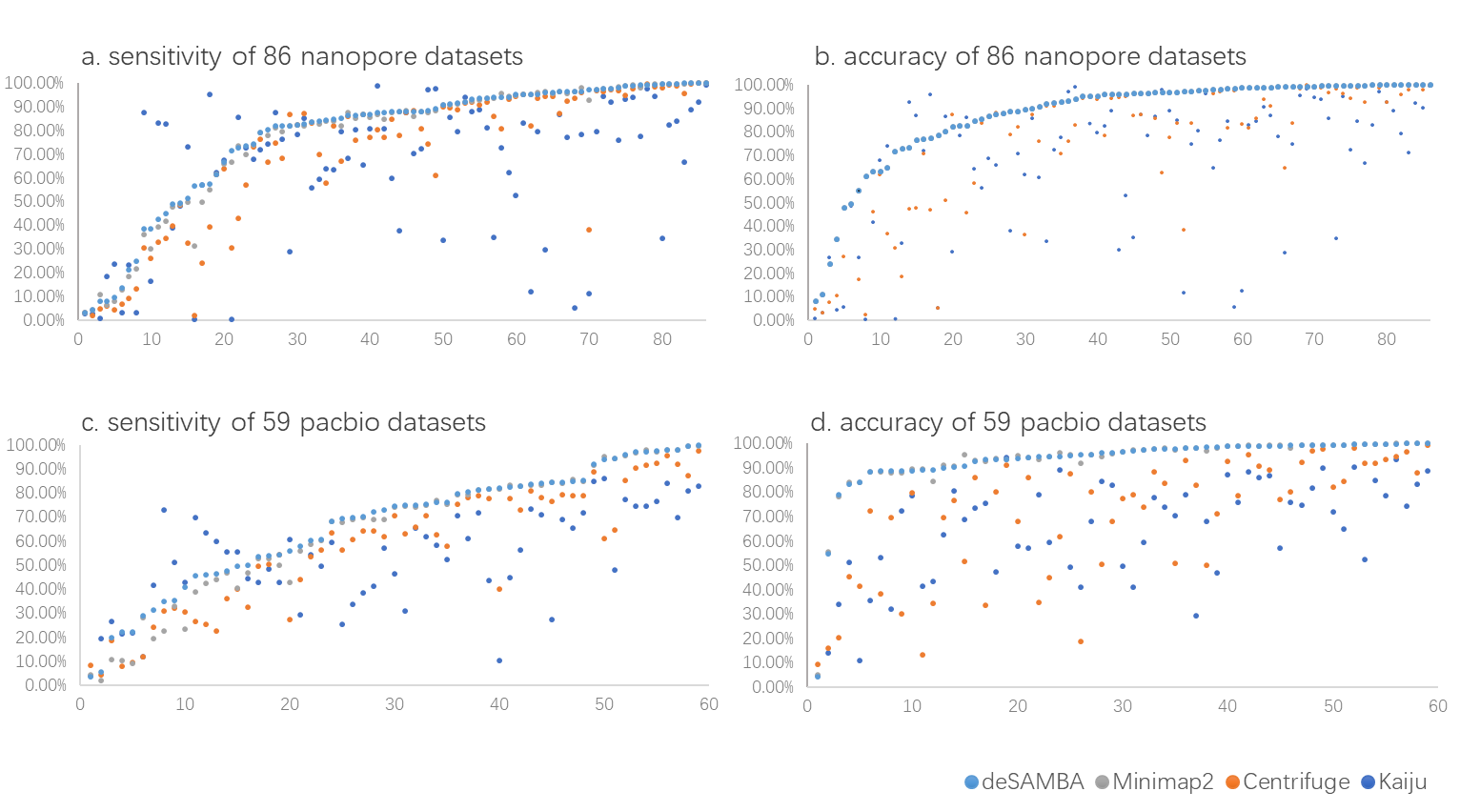


1. **The sensitivities and accuracies on the pseudo metagenomics datasets**

The sensitivities (subfigures a and c) and accuracies (subfigures b and d) of various approaches are shown by various colored dots (light blue, gray, orange and blue dots for the results of deSAMBA, Minimap2, Centrifuge and Kaiju, respectively).


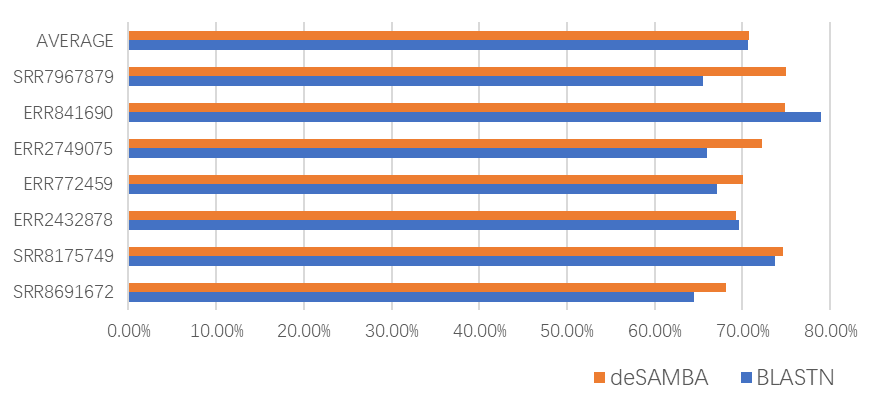


1. **The sensitivity of BLASTN on 7 WR-datasets with low sensitivity**

There are 7 WR-datasets (accession numbers: SRR7967879, ERR841690, ERR2749075, ERR772459, ERR2432878, SRR8175749 and SRR8691672) which deSAMBA had <80% sensitivities (indicated by orange bars in the figure). BLSATN was used to directly align the reads of these datasets to their ground truth genomes, respectively. The blue bars indicate the sensitivities of BLASTN on these datasets. The sensitivity of BLASTN for a given dataset is defined by the proportion of the reads having alignment outputs.

**Supplementary Notes**

- 1. Implementation of benchmark

All the benchmarks were carried out on a server with 4 Intel E7-4820 CPUs (32 cores) and 1 TB RAM running Ubuntu Linux OS. All the benchmarked classification tools were run in 8 CPU threads. Some detailed information about employed reference sequences, the real sequencing datasets and the command lines used for read classification is as following.

2.1 Reference sequences used for benchmark

We downloaded all reference sequences from NCBI RefSeq database. A genome sequence from RefSeq database was employed only if it is marked as a “complete genome”. There are totally 8621 bacterial, 251 archaea and 7412 viral genomes being used. The RefSeq ID and Taxonomy ID are described in “reference describe.txt”. For kaiju, the reference index was built using NCBI protein database, due to its specifically designed read classification approach.

Downloading “assembly_summary” files:

ftp://ftp.ncbi.nlm.nih.gov/genomes/refseq/bacteria/assembly_summary.txt

ftp://ftp.ncbi.nlm.nih.gov/genomes/refseq/viral/assembly_summary.txt

ftp://ftp.ncbi.nlm.nih.gov/genomes/refseq/archaea/assembly_summary.txt

Download “fna” file using “ftp_path” item of each assembly accession line. For example:

ftp://ftp.ncbi.nlm.nih.gov/genomes/all/GCF/000/189/935/GCF_000189935.1_ASM18993v2/GCF_000189935.1_ASM18993v2_genomic.fna.gz

All reference sequences were downloaded using an in-house script:

https://github.com/hitbc/deSAMBA-meta/blob/master/download

We used this script to download the reference sequences via the following commandlines:

bash ./download -P 10 -o $DOWNLOAD -d bacteria refseq

bash ./download -P 10 -o $DOWNLOAD -d viral refseq

bash ./download -P 10 -o $DOWNLOAD -d archaea refseq

All reference files were combined before the construction to the index:

find $DOWNLOAD/ -name "*.fna" | xargs -n 1 cat > ref.fa

2.2 Pseudo-metagenomics datasets and ground truth

We downloaded the 145 real sequencing pseudo-metagenomics datasets from NCBI Sequence Read Archive (SRA). The datasets are from various bacteria, viral or archaea genomes. It is also worthnoting that for all the datasets, only the reads longer than 1000 bp were used for the benchmark.

The ground truth of a dataset was got from NCBI SRA database, and the true organism of a dataset is shown in the “Organism” label:

https://trace.ncbi.nlm.nih.gov/Traces/sra/?run= [ SRA accession]

The taxonomy and genus labels of a dataset are got from NCBI taxonomy database:

https://www.ncbi.nlm.nih.gov/Taxonomy/Browser/wwwtax.cgi

2.3 Command lines used for read classification

1) Benchmark for deSAMBA (version 1.0)

Building index:

bash ./build-index ref.fa INDEX_DIR

Read classification:

deSAMBA classify -t 8 -s 62 INDEX_DIR read.fastq > classify.sam

2) Benchmark for minimap2[[1](#_ENREF_1)] (version 2.16 r922):

Building index for PACBIO data:

minimap2 -x map-pb -d ref_pb.mmi ref.fa

Building index for NANOPORE data:

minimap2 -x map-ont -d ref_np.mmi ref.fa

Read classification for PACBIO data:

minimap2 -a ref_pb.mmi read.fastq -t 8 > classify.sam

Read classification for NANOPORE data:

minimap2 -a ref_np.mmi read.fastq -t 8 > classify.sam

3) Benchmark for Centrifuge[[2](#_ENREF_2)] (version 1.0.4)

Building index:

centrifuge-build -p 16 --conversion-table ref.map --taxonomy-tree nodes.dmp --name-table names.dmp ref.fa INDEX_DIR

Read classification:

centrifuge-class INDEX_DIR --out-fmt sam -t -p 8 -U read.fastq > classify.cen

4) Benchmark for KAIJU[[3](#_ENREF_3)] (version 1.6.3)

Building index:

kaiju-makedb -s refseq

Read classification in MEM mode:

kaiju -t node.dmp -f kaiju_db_refseq.fmi -v -a mem -i read.fastq -z 8 > classify.kai

- 1. References

1. Li H. Minimap2: pairwise alignment for nucleotide sequences, Bioinformatics 2018;34:3094-3100.

2. Kim D, Song L, Breitwieser FP et al. Centrifuge: rapid and sensitive classification of metagenomic sequences, Genome Research 2016;26:1721-1729.

3. Menzel P, Ng KL, Krogh A. Fast and sensitive taxonomic classification for metagenomics with Kaiju, Nat Commun 2016;7:11257.
